# Knowledge and attitudes of healthcare workers in Chinese intensive care units regarding 2009 H1N1 influenza pandemic

**DOI:** 10.1186/1471-2334-11-24

**Published:** 2011-01-25

**Authors:** Xiaochun Ma, Zhenyang He, Yushan Wang, Li Jiang, Yuan Xu, Chuanyun Qian, Rongqing Sun, Erzhen Chen, Zhenjie Hu, Lihua Zhou, Fachun Zhou, Tiehe Qin, Xiangyuan Cao, Youzhong An, Renhua Sun, Xijing Zhang, Jiandong Lin, Yuhang Ai, Dawei Wu, Bin Du

**Affiliations:** 1 The First Affiliated Hospital of China Medical University, Shenyang, PR China; 2 Hainan Provincial People's Hospital, Haikou, PR China; 3 The Second Hospital of Jilin University, Changchun, PR China; 4 Fuxing Hospital, Capital Medical University, Beijing, PR China; 5 Beijing Tongren Hospital, Capital Medical University, Beijing, PR China; 6 The First Affiliated Hospital of Kunming Medical College, Kunming, PR China; 7 The First Affiliated Hospital of Zhengzhou University, Zhengzhou, PR China; 8 Ruijin Hospital, Shanghai Jiaotong University, Shanghai, PR China; 9 Hebei Medical University Fourth Hospital, Shijiazhuang, PR China; 10 The Affiliated Hospital of Inner Mongolia Medical College, Huhhot, PR China; 11 The First Affiliated Hospital, Chongqing Medical University, Chongqing, PR China; 12 Guangdong General Hospital, Guangzhou, PR China; 13 Affiliated Hospital of Ningxia Medical University, Yinchuan, PR China; 14 Peking University People's Hospital, Beijing, PR China; 15 Zhejiang Provincial People's Hospital, Hangzhou, PR China; 16 Xijing Hospital, Xi'an, PR China; 17 The First Affiliated Hospital of Fujian Medical University, Fuzhou, PR China; 18 Xiangya Hospital, Central South University, Changsha, PR China; 19 Qilu Hospital of Shandong University, Jinan, PR China; 20 Peking Union Medical College Hospital, Beijing, PR China

## Abstract

**Background:**

To describe the knowledge and attitudes of critical care clinicians during the 2009 H1N1 influenza pandemic.

**Methods:**

A survey conducted in 21 intensive care units in 17 provinces in China.

**Results:**

Out of 733 questionnaires distributed, 695 were completed. Three hundred and fifty-six respondents (51.2%) reported their experience of caring for H1N1 patients. Despite the fact that 88.5% of all respondents ultimately finished an H1N1 training program, only 41.9% admitted that they had the knowledge of 2009 H1N1 influenza. A total of 572 respondents (82.3%) expressed willingness to care for H1N1 patients. Independent variables associated with increasing likelihood to care for patients in the logistic regression analysis were physicians or nurses rather than other professionals (odds ratio 4.056 and 3.235, p = 0.002 and 0.007, respectively), knowledge training prior to patient care (odds ratio 1.531, p = 0.044), and the confidence to know how to protect themselves and their patients (odds ratio 2.109, p = 0.001).

**Conclusion:**

Critical care clinicians reported poor knowledge of H1N1 influenza, even though most finished a relevant knowledge training program. Implementation of appropriate education program might improve compliance to infection control measures, and willingness to work in a pandemic.

## Background

The novel 2009 influenza A (H1N1) attacked almost all countries since March 2009, which resulted in a severe global healthcare problem leading to the declaration of the first phase 6 global influenza pandemic by the World Health Organization on June 11, 2009.

Although the clinical manifestation remains mild to moderate for the initial 3 to 6 days [1-3], about 25% of patients experience rapid deterioration, leading to intensive care unit (ICU) admission within 1 day after hospitalization [1]. Based on a model simulating the potential impact of H1N1 influenza pandemic in the United States, Presanis and colleagues found that an autumn-winter pandemic wave of H1N1 with comparable severity per case could lead to approximately 40,000 - 140,000 ICU admissions (13 - 46 per 100,000 population) [4]. Moreover, Zilberberg et al estimated that 46 million people would contract the infection, resulting in 2.7 million hospitalizations, with 331,587 episodes of acute respiratory failure requiring mechanical ventilation, equivalent to an increase in the volume of mechanical ventilation of 23% to 45% over the current use [5]. Although the above estimates of the potential numbers of critically ill patients were crude at best, both suggested that, during the influenza pandemic, healthcare workers (HCWs) in ICUs should be prepared to provide critical care support for an excessive volume of critically ill patients over the course of several months. Therefore, it was strongly recommended by the Task Force for Mass Critical Care that, during a disaster, "hospitals with ICUs should plan and prepare to provide emergency mass critical care every day of the response for a total critically ill patients census at least triple usual ICU capacity" [6].

Several studies explored the knowledge and attitudes of HCWs towards transmissible diseases as well as the willingness to work during a pandemic, but most were conducted in hypothetical scenarios [7-9], with only one study examining the behaviors of critical care clinicians in an anticipated influenza pandemic [10]. A common finding of the above studies was that as many as 50% of HCWs reported that they would be unlikely to care for patients during a pandemic, which might even worsen the situation of workforce shortages especially when an excessive patient volume is anticipated.

The purpose of our survey was to assess the knowledge and attitudes of critical care clinicians in Chinese ICUs during the current influenza pandemic. We also tried to identify independent predictors of unwillingness to work, in order to formulate an effective strategy to improve the preparedness of HCWs.

## Methods

### Setting

This study was conducted in 21 adult ICUs in 17 provinces in China. Among the 21 ICUs, 20 were members of China Critical Care Clinical Trial Group (CCCCTG). CCCCTG is a collaborative research network that was established in January 2009, with 24 participating ICUs from 24 tertiary hospitals in 21 provinces. These ICUs had an average of 20.8 ± 14.1 beds (corresponding to 1.1 ± 0.5% of total hospital beds), 13.2 ± 10.6 intensivists, and 42.1 ± 32.1 ICU nurses.

### Survey Participants and Protocol

On December 25, 2009, a survey questionnaire in companion to an instruction was sent by e-mail to the contact persons of individual participating ICUs. The contact persons were asked to encourage as many as HCWs in their ICUs to participate the survey, by distributing the voluntary and anonymous survey questionnaire in electronic format.

A reminder was e-mailed to all contact persons 2 weeks after the first mailing. The contact persons were required to collect all questionnaires and send back by e-mail before January 15, 2010. Any critical care clinicians not responding after the deadline were regarded as non-respondents.

Our study was approved by the institutional review board of Peking Union Medical College Hospital.

### 2009 H1N1 influenza pandemic training program

As a response to the 2009 H1N1 influenza pandemic, all hospitals were required by local healthcare authorities to provide training programs to all hospital staff via seminars. These training programs were mainly 2 to 3-hour lectures, developed based on the guidelines issued by Ministry of Health, often involving diagnosis, treatment, and infection control related to 2009 H1N1 influenza [11,12]. There was no posttest to evaluate the extent of information attainment by the attendees.

### Survey questionnaire

Based on the study of Daugherty and colleagues [10], a 36-item survey questionnaire was designed to assess the knowledge and attitudes of critical care clinicians related to the current 2009 H1N1 influenza pandemic (see Additional File 1). Data on the demographic characteristics of respondents, including age, sex, marital status, living status, status of influenza vaccination, and profession, were recorded. The professional status of the respondents was categorized as physicians, nurses, and others (including respiratory therapists, student nurses, and nurse assistants). The respondents were asked to report their experience of caring for H1N1 patients, as well as relevant training. They were also required to report the level of knowledge and the level of confidence in their ability to protect themselves and their patients from exposure to influenza at work. A 5-point Likert scale (complete agree, agree, neither agree nor disagree, disagree, and complete disagree) was used to elicit preferred answers. Finally, the respondents were asked to report their willingness to care for H1N1 patients.

### Statistical Analysis

We described clinicians' characteristics as continuous (age) or categorical variables (sex, marital status, living status, status of influenza vaccination, and profession). All Likert-scale responses were dichotomized into complete agree/agree versus neither agree nor disagree/disagree/complete disagree, and expressed in proportions.

Continuous variables were compared with the use of the Student's t-test or Mann-Whitney test. The chi-square test or Fisher's exact test was used to compare categorical variables. For determination of independent predictors for willingness to care for H1N1 patients, odds ratio (OR) was estimated on the basis of multivariate logistic regression analysis. Variables including clinicians' characteristics, knowledge, and attitudes were added into the model using stepwise conditional forward entry, if p < 0.1 in univariate analysis. An OR of less than 1 was associated with less likelihood to care for H1N1 patients, while an OR of greater than 1 was associated with more likelihood to care for H1N1 patients.

## Results

### Characteristics of respondents

A total of 733 questionnaires were distributed, and 695 were completed (89.9% response rate). An average of 33.1 ± 23.8 critical care clinicians responded to the survey in every participating ICU (median 27, interquartile range 18 to 40, range 6 to 100). Respondent characteristics were described in table 1.

**Table 1:** Respondent characteristics.

Characteristics	Physicians (n = 235)	Nurses (n = 435)	Others (n = 25)	Total (n = 695)
Male sex**	134 (57.0)	26 (6.0)	6 (24.0)	166 (23.9)
Age**	36.7 ± 6.6	28.1 ± 5.5	40.6 ± 12.5	30.4 ± 7.0
Married**	177 (75.3)	189 (43.4)	15 (60.0)	381 (54.8)
Living status				
With parents*	55 (23.4)	133 (30.6)	3 (12.0)	191 (27.5)
With children**	83 (35.3)	54 (12.4)	8 (32.0)	145 (20.9)
With spouse only	46 (19.6)	90 (20.7)	7 (28.0)	143 (20.6)
Alone**	46 (19.6)	149 (34.3)	6 (24.0)	201 (28.9)
NA	5 (2.1)	9 (2.1)	1 (4.0)	15 (2.2)
Vaccination for seasonal influenza	10 (4.3)	24 (5.5)	3 (12.0)	37 (5.3)
Vaccination for 2009 H1N1 influenza*	112 (47.7)	242 (55.6)	17 (68.0)	371 (53.4)

NA: not available

*p < 0.05, **p < 0.01 across groups by Chi-square test or Fisher exact test

Data are expressed as mean ± standard deviation (SD), or n (%)

There were more males among physicians (57.0%) compared to nurses (6.0%) and other professionals (24.0%). Significantly more nurses were single, living with their parents or living alone.

Only 37 (5.3%) of all respondents reported to receive vaccination for seasonal influenza during the current influenza season. In comparison, more than half were vaccinated for 2009 H1N1 influenza. Among those not receiving vaccination for 2009 H1N1 influenza, concern about vaccine safety (185, 57.1%) was the most common reason, followed by concern about vaccine efficacy (145, 44.8%), belief that H1N1 influenza is a mild disease in most patients (138, 42.6%), and contraindication for vaccination (53, 16.4%). Only 19 respondents (5.9%) reported that they did not have the access to H1N1 vaccines.

### Knowledge of risks and protection strategy of 2009 H1N1 influenza

Three hundred and fifty-six respondents (51.2%) reported experience caring for H1N1 patients, among whom 305 (85.7%) finished the H1N1 training program before caring for H1N1 patients (table 2). Despite the fact that 88.5% of all respondents ultimately finished the H1N1 training program, only 41.9% admitted that they had adequate knowledge of 2009 H1N1 influenza. Apart from the training program organized by hospital or local healthcare authorities, various media (including internet, television, and newspaper) were also major sources of knowledge. Moreover, significantly more physicians (60.9%) obtained relevant knowledge from medical journals compared with nurses (33.8%) and other professionals (8.0%) (table 2).

**Table 2:** Knowledge of 2009 H1N1 influenza among respondents.

Question, response	Physicians (n = 235)	Nurses (n = 435)	Others (n = 25)	Total (n = 695)
I have the experience caring for H1N1 patients	123 (52.3)	219 (50.3)	14 (56.0)	356 (51.2)
I have finished H1N1 training program**	220 (93.6)	383 (88.0)	12 (48.0)	615 (88.5)
I have finished H1N1 training program before I cared for H1N1 patients*	131 (55.7)	240 (55.2)	5 (20.0)	376 (54.1)
I had the knowledge of H1N1 influenza**,#	143 (60.9)	147 (33.8)	1 (4.0)	291 (41.9)
Sources of knowledge				
Television**	124 (52.8)	320 (73.6)	20 (80.0)	464 (66.8)
Newspaper**	105 (44.7)	269 (61.8)	19 (76.0)	393 (56.5)
Internet**	171 (72.8)	291 (66.9)	5 (20.0)	467 (67.2)
Scientific journal**	143 (60.9)	147 (33.8)	2 (8.0)	292 (42.0)
Education**	205 (87.2)	388 (89.2)	9 (36.0)	602 (86.6)
Other	6 (2.6)	14 (3.2)	0 (0)	20 (2.9)
I am confident that I understand the risks of a pandemic for patients and HCWs#	198 (84.3)	344 (79.1)	17 (68.0)	559 (80.4)
I am confident that I know how to protect myself and my patients during a pandemic#	190 (80.9)	326 (74.9)	18 (72.0)	534 (76.8)

*p < 0.05, **p < 0.01 across groups by Chi-square test or Fisher exact test

#The percentages reflect a complete agree or agree response to each question.

Data are expressed as n (%)

Significant associations between knowledge training and level of knowledge, as well as degree of confidence regarding protection were observed. Among 615 respondents who finished the H1N1 training program, 279 (45.4%) reported to have extensive knowledge, and 490 (79.7%) expressed confidence in their ability to protect themselves and their patients, compared with 10 (13.2%, p < 0.001) and 41 (53.9%, p < 0.001) out of 76 respondents without knowledge training. A similar association was also observed between finishing the training program before caring for patients (advanced training) and level of confidence in self-protection. In particular, 316 out of 376 respondents who finished advanced training were confident of self-protection, compared with only 218 out of 319 respondents without advanced training (80.4% vs. 68.3%, p < 0.001).

### Predictors of willingness to care for H1N1 patients

A total of 572 respondents (82.3%) expressed willingness to care for H1N1 patients. The most common reasons for unwillingness to care for H1N1 patients included concern about infection of family members (61/110, 55.5%) and themselves (33/100, 33.0%). Univariate analysis found no difference between respondents in their willingness to care for H1N1 patients, based on sex, age, marital status, and living status. However, professionals other than physicians and nurses expressed less willingness to care for H1N1 patients, while finishing the H1N1 training program (especially before caring for H1N1 patients), and the level of confidence to know how to protect themselves and their patients significantly increased the likelihood for respondents to care for H1N1 patients (table 3). Moreover, the vaccination for 2009 H1N1 influenza did not influence the willingness to care for H1N1 patients.

**Table 3:** Predictors of willingness to care for H1N1 patients: univariate analysis.

	Unwillingness to care for H1N1 patients (n = 121)	Willingness to care for H1N1 patients (n = 572)	p
Male sex	26 (21.5)	140 (24.5)	0.560
Age	30.6 ± 7.7	30.4 ± 6.8	0.774
Married	67 (55.4)	313 (54.7)	0.976
Living status			
With parents	26 (21.5)	164 (28.7)	0.134
With children	29 (24.0)	116 (20.3)	0.434
With spouse only	25 (20.7)	118 (20.6)	0.908
Alone	39 (32.2)	161 (28.1)	0.429
Profession			
Physicians	33 (27.3)	201 (35.1)	0.120
Nurses	77 (63.6)	357 (62.4)	0.881
Others	11 (9.1)	14 (2.4)	0.001
I have the experience caring for H1N1 patients	55 (45.5)	299 (52.3)	0.207
I have finished H1N1 training program	100 (82.6)	513 (89.7)	0.041
I have finished H1N1 training program before I cared for H1N1 patients	50 (41.3)	324 (56.6)	0.003
I am confident that I understand the risks of a pandemic for patients and HCWs#	91 (75.2)	466 (81.5)	0.147
I am confident that I know how to protect myself and my patients during a pandemic#	76 (62.8)	456 (79.7)	<0.001
Vaccinated for seasonal influenza	8 (6.6)	29 (5.1)	0.504
Vaccinated for 2009 H1N1 influenza	64 (52.9)	307 (53.7)	0.956

#The percentages reflect a complete agree or agree response to each question.

Data are expressed as mean ± standard deviation (SD), or n (%)

Logistic regression analysis revealed that physicians or nurses rather than other professionals, advanced training, and the confidence to know how to protect themselves and their patients were all independent variables associated with more likelihood to care for patients (table 4).

**Table 4:** Predictors of willingness to care for H1N1 patients: logistic regression analysis.

Variable	Odds Ratio	95%Confidence Interval	p
Profession			
Others	Reference		
Physician	4.056	1.663 - 9.889	0.002
Nurses	3.235	1.385 - 7.560	0.007
Finishing H1N1 training program before caring for patients	1.531	1.012 - 2.316	0.044
Confident to know how to protect themselves and their patients	2.109	1.366 - 3.257	0.001

## Discussion

This study demonstrated that, during the H1N1 influenza pandemic, only 40% of critical care clinicians reported to have extensive knowledge of 2009 H1N1 influenza, even though almost 90% of them received relevant training. In addition, about 82% of respondents were willing to care for H1N1 patients. Independent predictors of willingness to care for patients included profession, knowledge training before patient care, and level of confidence to protect themselves and their patients.

Although there had been several survey studies regarding the knowledge of and attitudes towards influenza or other transmissible diseases among HCWs [7-10], all were conducted before 2009 H1N1 influenza pandemic or in the setting of hypothetical case scenarios. Only one of these was performed among critical care clinicians [10]. Our study differed from previous studies because it was conducted during the H1N1 influenza pandemic, when more than half of the respondents reported experience caring for H1N1 patients. Therefore, we believe that the results of our study might more likely represent the actual response of HCWs.

Our study revealed that, even after significant training efforts, only 40% of HCWs reported to have extensive knowledge of H1N1 influenza. Lack of knowledge with regard to influenza pandemic was a common finding in previous studies [13,14], because the general public felt that infection control behaviors might do more harm than good and/or were unnecessary [15]. Some investigators believed that people were more likely to implement the recommended behaviors in the event of perceived personal risk [14,16]. However, our study suggested that this might not be the case. Even in the phase 6 global influenza pandemic, about 60% of HCWs working in ICUs (where the risk of secondary transmission of respiratory viruses might be particularly high [17-19]) realized that they lacked the necessary knowledge. Similar to the study of Daugherty et al [10], our study suggested major gaps between HCWs' knowledge and recommended infection control measures, representing a serious public health concern.

About 18% of critical care HCWs in our study reported that they were unlikely to work during a pandemic, a proportion similar to that reported by Daugherty [10]. In comparison, previous studies found that significantly fewer HCWs might commit to work in the event of a transmissible disease outbreak. However, it should be pointed out that these studies were carried out among general HCWs rather than critical care clinicians. For example, in a survey of 303 employees at 3 health departments in Maryland from March to July 2005, 163 (53.8%) indicated they would likely report to work in the event of an influenza pandemic [7]. Moreover, Syrett and colleagues reported that even fewer HCWs (18%) in University of Rochester Medical Center committed to work in the hypothetical setting of a transmissible infectious agent with only unproven, experimental prophylaxis or treatment [8]. Both the present study and Daugherty's study found that significantly more critical care clinicians reported willingness to work during an influenza pandemic [10]. Similarly, Martinese et al found that, in comparison with the general HCWs, staff working in emergency and acute medical wards directly responsible for the care of influenza patients were more likely to report for duty [9]. The reason for such a significant difference between critical care clinicians and general HCWs remains to be elucidated, but could be related to the perception of the importance of their roles in response to a public health crisis [7,9].

In addition, the psychosocial impact of a pandemic on HCWs can be significant and multifaceted. They might be frightened for both their own and their families' health, and experience significant psychosocial stress. Our study supported the above findings. Among 110 respondents who were unwilling to care for H1N1 patients, 90% expressed great concern about the possibility of contracting and/or transmitting H1N1 influenza to their family and themselves.

The univariate analysis found that knowledge training was associated with better understanding of H1N1 influenza, and more confidence to protect themselves and their patients as well. The logistic regression analysis also demonstrated that knowledge training before patient contact was an independent factor associated with willingness to care for H1N1 patients. All these data suggested that implementation of an educational intervention might result in better preparedness of critical care HCWs for an H1N1 influenza pandemic. This conclusion was also supported by studies which showed that education programs might lead to higher compliance to infection control measures such as hand hygiene [20], and more importantly, the reduction of multi-resistant bacteria in hospital settings [21]. Other studies had suggested that the implementation of appropriate education and protective measures improved willingness to work [8,22], although investigators did not have the chance to test this hypothesis during the H1N1 influenza pandemic.

We reported that only half of all respondents received vaccination for 2009 H1N1 influenza. This finding was consistent with previous studies suggesting that HCWs were reluctant to vaccinate themselves [23,24] and their children [25]. These studies also demonstrated that, like our results, the major reason for refusing the pandemic vaccine was safety concerns, especially when people believed that the vaccine went through an accelerated approval procedure [23,25,26]. Other reasons included concerns about efficacy, and the belief "that H1N1 is a mild disease" [25,26]. These studies also suggested that the willingness to accept the pandemic H1N1 influenza vaccine might be improved by promotion of vaccination for seasonal influenza, and recognition of the role of HCWs in the transmission of influenza to patients and even family members [26].

Our study was subject to a number of limitations. First, we could not exclude the possible impact of selection bias. However, the high response rate (89.9%) indicated that the study sample represented the actual hospital staff in participating ICUs. Such a high response rate was achieved through the great effort of contact persons in individual ICUs, such as personal distribution of the survey questionnaire to all ICU staffs, and encouraging all potential respondents to complete the questionnaire and return it before the deadline. Second, our study was conducted in 21 tertiary hospitals, possibly limiting generalization of findings to other hospitals. Nevertheless, local healthcare authorities in China required that all severe cases of H1N1 influenza should be transferred to tertiary hospitals. As a result, this study was conducted in hospital settings where most severe cases of H1N1 influenza were likely to be treated. Last, previous studies suggested that self-reported practice might not represent the actual practice [27]. We might deduce that the actual compliance to infection control measures would be even lower.

## Conclusion

In conclusion, this study raised important concern about the adequacy of knowledge even during the H1N1 influenza pandemic. There is a clear need for knowledge training programs, in order to improve the understanding of the risks and prevention strategies among critical care clinicians, which should in turn improve the confidence of clinicians to provide the right care to their patients and protect themselves as well.

## Competing interests

The authors declare that they have no competing interests.

## Authors' contributions

BD conceived of the study, participated in its design and coordination, had full access to all of the data in the study and takes responsibility for the integrity of the data and the accuracy of the data analysis. MC, ZYH, and YW conceived of the study, and participated in its design and coordination, and helped to draft the manuscript. LJ, YX, CQ, RS, EC, ZJH, LZ, FZ, TQ, XC, YZA, RS, XZ, JL, YHA, and DW participated in the design of the study, and helped to draft the manuscript. All authors reviewed and approved the final manuscript.

## Pre-publication history

The pre-publication history for this paper can be accessed here:

http://www.biomedcentral.com/1471-2334/11/24/prepub

## Supplementary Material

Additional file 1 **Survey Questionnaire**. Survey questionnaire: Knowledge and attitudes of critical care clinicians regarding 2009 H1N1 influenza pandemic Click here for file
